# Cost-effectiveness of different treat-to-target strategies in rheumatoid arthritis: results from the DREAM registry

**DOI:** 10.1186/s41927-019-0064-9

**Published:** 2019-04-29

**Authors:** Celine J. van de Laar, Martijn A. H. Oude Voshaar, Harald E. Vonkeman

**Affiliations:** 1 Arthritis Center Twente, Medisch Spectrum Twente, University of Twente, Enschede, The Netherlands; 2 Transparency in Healthcare b.v., Hengelo, The Netherlands; 3 Department of Psychology, Health & Technology, University of Twente, Enschede, The Netherlands; 4 Department of Rheumatology and Clinical Immunology, Medisch Spectrum Twente, Enschede, The Netherlands

**Keywords:** Cost-effectiveness, Markov, Modelling, Rheumatoid arthritis, Treat-to-target, Probabilistic sensitivity analysis

## Abstract

**Background:**

Adjusting medication of patients with rheumatoid arthritis (RA) until predefined disease activity targets are met, i.e. Treat-to-Target (T2T), is the currently recommended treatment approach. However, not much is known about long-term cost-effectiveness of different T2T strategies.

We model the 5-year costs and effects of a step-up approach (MTX mono - > MTX + csDMARD combination - > Adalimumab - > second anti-TNF) and an initial combination therapy approach (MTX + csDMARD - > MTX + csDMARD higher dose - > anti-TNFs) from the healthcare and societal perspectives, by adapting a previously validated Markov model.

**Methods:**

We constructed a Markov model in which 3-monthly transitions between DAS28-defined health states of remission (≤2.6), low (2.6 < DAS28 ≤ 3.2), moderate (3.2 < DAS28 ≤ 5.1), and high disease activity (DAS28 > 5.1) were simulated. Modelled patients proceeded to subsequent treatments in case of non-remission at each (3-month) cycle start. In case of remission for two consecutive cycles medication was tapered, until medication-free remission was achieved. Transition probabilities for individual treatment steps were estimated using data of Dutch Rheumatology Monitoring registry Remission Induction Cohort I (step-up) and II (initial combination). Expected costs, utility, and ICER after 5 years were compared between the two strategies. To account for parameter uncertainty, probabilistic sensitivity analysis was employed through Gamma, Normal, and Dirichlet distributions. All utilities, costs, and transition probabilities were replaced by fitted distributions.

**Results:**

Over a 5-year timespan, initial combination therapy was less costly and more effective than step-up therapy. Initial combination therapy accrued €16,226.3 and 3.552 QALY vs €20,183.3 and 3.517 QALYs for step-up therapy. This resulted in a negative ICER, indicating that initial combination therapy was both less costly and more effective in terms of utility gained. This can be explained by higher (±5%) remission percentages in initial combination strategy at all time points. More patients in remission generates less healthcare and productivity loss costs and higher utility. Additionally, higher remission percentages caused less bDMARD use in the initial combination strategy, lowering overall costs.

**Conclusion:**

Initial combination therapy was found favourable over step-up therapy in the treatment of Rheumatoid Arthritis, when considering cost-effectiveness. Initial combination therapy resulted in more utility at a lower cost over 5 years.

**Electronic supplementary material:**

The online version of this article (10.1186/s41927-019-0064-9) contains supplementary material, which is available to authorized users.

## Background

Rheumatoid arthritis (RA) is a systemic autoimmune disease with alternating periods of lower and higher disease activity. RA may have a chronic, progressive course, leading to functional impairment and reduced quality of life [1]. The main objective of treatment is to achieve suppression of inflammation as soon as possible, to minimize symptoms in the short-term and to retard progression of structural damage in the long term. The adoption of modern treatment strategies, together with new and expensive, biological or targeted synthetic, disease-modifying anti-rheumatic drugs (DMARDs) have considerably improved patient outcomes. However, the cost of these new drugs combined with the lifelong scope of RA treatment has resulted in a considerable cost burden on payers of healthcare costs [2].

The approach currently recommended for RA treatment involves titrating medication dosages until pre-specified disease activity targets (either remission and low disease activity (LDA) or LDA) have been met and maintaining these targets over time. Such so-called treat to target strategies (T2T) have proven to be more effective and to generate more utility than usual care [3, 4]. A previous study by Vermeer et al. found that the focus on rapid suppression of inflammation results in high initial costs, but has been shown to be well within willingness-to-pay thresholds in the long run [3]. The European League Against Rheumatism (EULAR) and American College of Rheumatology (ACR) have developed comprehensive recommendations on the setup and implementation of T2T in clinical practice [5, 6]. However, even when following these guidelines, different treatment strategies can be adopted, for example T2T protocols employing step-up therapy, initial combination therapy or initial biological DMARDs therapy. These differences may lead to important variation in clinical outcomes, costs, and utility.

Not much is known about real world cost-effectiveness of such alternatives however, because multiple previous health-economic evaluations of RA treatment strategies focus on the optimal place of one particular drug in some sequence of treatment options [7]. Moreover, models are typically fed with data from clinical trials with selected patient populations. This limits the generalizability of the results and could potentially wrongly estimate real-world treatment effectiveness for various reasons, such as selection criteria of trials favouring patients likely to respond, and wash-out period before treatment initiation [8].

The aim of this paper is to compare the long-term cost-effectiveness of step-up therapy and initial combination therapy from a societal perspective, by expanding on a previously validated Markov modelling approach and populating the model using data extracted from two real world cohorts of unselected RA patients that have been treated using the respectively modelled strategies. This will allow to improve the conceptual model framework for RA which can be used in the future for more comparisons of treatments and strategies.

## Methods

### Health economic modelling

This study used a Markov model to assess long term cost-effectiveness of two T2T strategies in treatment of rheumatoid arthritis [9]. Step-up therapy was compared to initial combination therapy in a model based health economic evaluation in which economic consequences of two treatment strategies are evaluated within a mathematical framework. In accordance with the ISPOR principles [10] for good practice for decision analytic modelling in healthcare evaluation, all model input for our study was derived directly from the various DREAM cohorts, as described in the next section.

### Data

All data used in this study was derived from two real world observational studies in which patients were treated according to T2T protocols, both aiming at achieving 28 joint-count disease activity score (DAS28) remission (i.e. DAS28 score ≤ 2.6) in order to extrapolate over a period of 5 years. Baseline characteristics of the patients are summarized in the Additional file 1.

**Table 1:** Base Analysis results

	Step-up	Initial Combination
Mean costs (€)	25,377.01	20,856.56
Mean utility (QALY)	3.501	3.545
ICER	–	−139,000 (Dominating)

Outcomes and costs were registered in the same way in both cohorts and data collection, including DAS28-assessment, was carried out by trained rheumatology nurses. Patients were included upon diagnosis with early-onset moderate to severe RA (DAS28 > 3.2) and were DMARD-naïve. For both cohorts, data was collected in the same hospitals.

Patients in Remission Induction Cohort I (RIC I) (step-up therapy) were initially treated with methotrexate (MTX) monotherapy, followed by addition of sulfasalazine. In case of persistent moderate disease activity (moderate or high; DAS28 > 3.2) sulfasalazine could be replaced by TNFi. Due to reimbursement policies in the Netherlands, patients with DAS28 > 3.2 were allowed to start TNFi’s. Patients were evaluated at baseline, 8, 12, 20, 24, 36, and 52 weeks, and every 3 months thereafter [11]. Consecutive patients entered this cohort between 2006 and 2012 and were followed regularly thereafter.

Patients in Remission Induction Cohort II (RIC II) (initial combination) were initially treated with combination csDMARD therapy, followed by high-dose combination therapy. In case of persistent moderate to high disease activity (> 3.2), a TNFi could be started, replacing one of the csDMARDs. Patients were assessed at baseline, 2, 4, 6 months and every 3 months thereafter [12]. Consecutive patients entered this cohort from 2012 onward and were followed regularly thereafter.

Patients in both cohorts have given written informed consent before inclusion. The attending physician and the patients were advised to follow the per-protocol predefined assessments and treatment decisions. Treatment changes could be made at any time point at the discretion of the rheumatologist. In general, conformity to the protocol was good [13].

### Markov model

Markov models can synthesize different types of costs and outcomes (utility, effectiveness) over a specified time [14–16]. For this study, using the data from DREAM RIC I and II, 5-year outcomes of two T2T-strategies were simulated using such a Markov model. Models consist of a finite number of ‘Markov states’ through which modelled patients move, with the probability to move from one state to another depending on transition probabilities. Different costs and outcomes accrue and lead to the eventual quality adjusted life years and healthcare costs, depending on the Markov states and treatments modelled patients move through.

The Markov model used in this study expands on a model introduced by Welsing et al. who also showed that the model has predictive validity in RA [15, 17]. In the present study patients are always in one of four mutually exclusive disease activity related health states. The health states are defined by the commonly used disease activity score in 28 joints (DAS28): remission (DAS28 ≤ 2.6), low disease activity (2.6 < DAS28 ≤ 3.2), moderate disease activity (3.2 < DAS28 ≤ 5.1), and high disease activity (DAS28 > 5.1) [18]. A time horizon of 5 years was used. This time horizon is divided into 20 cycles of 3 months and modelled patients may shift from one health state to another at the start of each cycle, with the transition probabilities depending on their health state at the beginning of the cycle and the medication they are using at the start of that cycle. Due to the fact that in real life, transitions are not automatically expected to occur at the beginning of a 3-monthly cycles, within-cycle correction was applied. Patients could move to a different health state at any point in that cycle. This method corrects cycle rewards and cost overestimation by considering the percentage of patients in each health state at the beginning and end of the cycle.

**Fig. 1:**
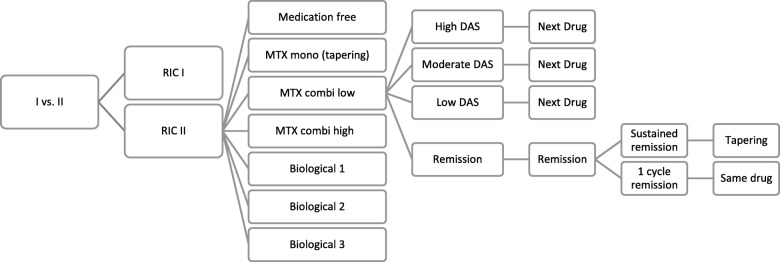
This figure represents the Markov Structure of the initial combination strategy. MTX combi low: low dose csDMARD combination therapy; MTX combi high: High dose csDMARD combination therapy; Biological 1,2,3: bDMARD therapy. High DAS: High DAS28, Moderate DAS: Moderate DAS28; Low DAS: Low DAS28; Next Drug: patients move downstream; Remission: in case of sustained remission medication will be tapered, in case of only 1 cycle in remission, patient continues the same treatment

All patients initially enter the model on the first medication of their treatment protocol (resp. MTX monotherapy (RIC I) or MTX combination therapy (RIC II)). See Fig. 1. After the first cycle, patients will either be in remission (DAS28 ≤ 2.6) and stay on the same drug for another cycle, or not in remission (DAS28 > 2.6) and progress to the next drug as prescribed by the protocol. When patients sustain remission for two cycles (6 months), their medication will be tapered, as specified by the protocols. They will move to the preceding drug, or a medication-free state, in case no more preceding drugs are available and if their remission sustains for an adequate amount of time. For example, a patient that sustains remission on MTX monotherapy (in RIC I) for 2 cycles, will ‘jump’ to low-dose MTX. If the patient sustains remission for another six months, he/she will ‘jump’ to the medication-free state. Modelled patients will move one medication step up in case of a flare (DAS28 > 2.6), until it the flare under control. Model input.

Transition matrices for each treatment step were derived from data obtained from the subpopulation of patients treated with the relevant medication and dosages. Chi-squared statistics tests were used to verify the stability over time of the obtained transition probabilities [19]. All DAS-28 measures obtained during the period of time patients were treated using medication and dosages relevant to a treatment step were used to estimate 3 monthly transition probabilities from the sample proportions [20, 21]. Since clinic visits were not always scheduled in exact 3 month increments, a range of 1.5 months was used for DAS28 measurement moments. The distribution of patients of the four DAS28 states will be compared to observed daily clinical practice outcomes in RIC I.

The EuroQol five dimensional (EQ-5D) [22] questionnaire was used to value the quality of life in all four respective health states. The EQ-5D was recorded during all clinical visits. Utility scores of patients in each of the four DAS28 states were averaged. The questionnaire assesses a patients well-being on five dimensions: mobility, self-care, daily activities, pain/discomfort and anxiety/depression. Each dimension is valued using three levels: no problems, some problems, or extreme problems. The EQ-5D disserts 3^5^ = 243 different states of health. Each state is valued to create a utility score between 0 and 1 using the Dutch EQ-5D tariff [23]. A score of 0 represents a state that is as desirable as death. A score of 1 is considered perfect health. The average EQ-5D utility score of observed patients in each of the four DAS28 health states is used in the model.

To accurately reflect the costs that are connected to specific health states, both healthcare consumption and cost of absence from paid labour are included. A healthcare consumption questionnaire with questions about the type and amount of different care ‘units’ patients have received since their last appointment with the rheumatologist was used. This includes appointments with any type of specialist, general physician, or use of other types of care or medication. These care units were multiplied using the 2016 updated price index numbers to calculate the average amount of care consumption per health state [24]. Average sick days are measured as the average number of days per 3 months that patients have reported sick to their employers. It was assumed that the cost of one employee not being able to work for 1 day is €230, based on a report by The Netherlands Organisation for applied scientific research in 2014 [25]. To account for the fact that not all patients have paid jobs, the proportion of paid jobs patients (split by age and sex) is multiplied with average sick days. Leading to

1 $$ {{\mathrm{SD}}_{\mathrm{ij}}}^{\ast }\ \mathrm{workforce}\ {\mathrm{participation}}^{\ast }\ 230+{\mathrm{ZoCo}}_{\mathrm{ij}} $$

(i and j respectively refer to health state and cohort) for total health state-specific cost in both cohorts. Where SD refers to number of sick days per state and ZoCo refers to the Dutch Healthcare Consumption Questionnaire (HCQ).

#### Outcomes

All outputs in the model are globally discounted annually, using 4% for cost and 1.5% for effectiveness, as recommended in the Dutch Guideline for Economic Evaluations in Healthcare [24]. The primary outcome measure was the incremental cost effectiveness ratio (ICER). i.e., the incremental cost for one additional quality adjusted life year (QALY). In the Netherlands, the generally accepted threshold below which treatments are considered cost-effective lies between €20,000 and €100,000. In this paper, both extremities will be considered.

2 $$ ICER=\frac{\Delta Cost}{\Delta Effectiveness}=\frac{C_{II}-{C}_I}{E_{II}-{E}_I} $$

where the subscripts I and II refer to the compared interventions.

#### Monte Carlo simulation

Five thousand patients were simulated individually using Monte Carlo simulation, which allows to keep track of the disease course of each modelled patient as they moved through different cycles. The model was constructed and analysed using TreeAge Pro software (Williamstown MA, USA). The software assigns and records all Markov states, transitions, and costs and outcomes to each individual patient.

### Probabilistic sensitivity analysis

To account for uncertainty in the parameter estimates, distributions for all cost and utility estimates were fitted. Different distributions were evaluated in order to find the most appropriate ones for these parameters. Different suggestions and guidelines were evaluated [26–28]. These included normal, gamma, logistic, beta, and Poisson distributions. Using Anderson Darling/SK and Chi^2 tests, the most appropriate distributions were selected. The transition probabilities also face a level of uncertainty. All transition probability matrices have been re-specified using Dirichlet distributions. The matrices that included less data will incorporate more uncertainty than those with higher numbers of observations. In the probabilistic sensitivity analysis, 2000 × 200 runs of the model were performed where the input parameters were re-sampled from these distributions for each iteration.

## Results

### Comparison of model predicted- and clinically observed disease activity outcomes over 5 years

**Fig. 2:**
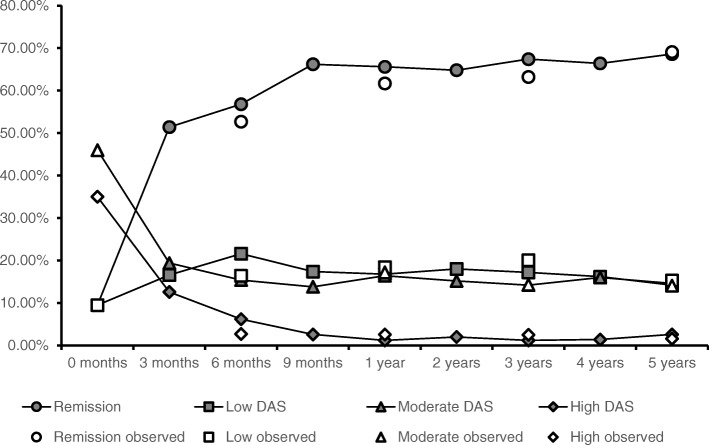
Distribution of simulation cohort over DAS-28 states, compared to observed distributions of RA patients in RIC I. Observed data is available at baseline (corresponds to baseline distribution for modelled patients), 6 months, 1 year, 3 years and at 5 years. DAS: DAS-28 state, Remission observed: percentage of observed patients in remission, low observed: percentage of observed patients in low DAS-28 state, moderate observed: percentage of observed patients in moderate DAS-28 state, high observed: percentage of observed patients in high DAS-28 state

Figure 2 presents the distribution of the modelled patients over the four DAS28 states for each of the twenty 3-monthly cycles, compared with the distribution of patient over the disease activity states as actually observed in daily clinical practice in RIC I [11]. The percentage of modelled patients in remission increases from ~ 10% at baseline to about 65% at the 1 year visit, which closely corresponds to the remission percentages seen in the cohort in the actual patients. Similar results can be seen for the other disease activity states, which supports the validity of the projected disease activity outcomes that are generated by the model. Comparison yields similar results when comparing the result of the initial combination strategy with result from RIC II.

### Base analysis

Results from the base analysis are shown in Table 1. The ICER of initial combination therapy versus step-up therapy with 5000 simulated patients is − 139.000. Initial combination therapy is dominant over step-up therapy. In this case, initial combination therapy is less costly (€20,856.56 vs €25,377.01) and more effective (3.54 vs 3.50 QALY) over 5 years. This indicates that initial combination therapy is cost-effective and dominates step-up therapy (Table 1).

### Probabilistic sensitivity analysis

**Fig. 3:**
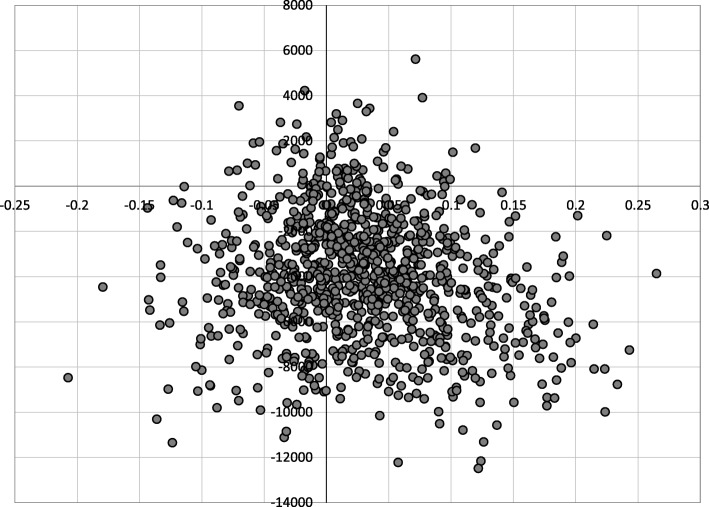
Cost-effectiveness plane of the comparison of initial combination therapy with step-up therapy. Results of probabilistic sensitivity analysis. Five hundred patients are displayed. X-axis: incremental effects (in Quality-Adjusted Life Years). Y-axis: Incremental costs (in €‘s). 64% of trials are in the southeast quadrant, and thus cost-saving. 87% of trials are cost-effective at a Willingness-to-Pay threshold of €60,000

Table 2 depicts the results of the PSA. Two thousand samples of 200 patients were run. The results show that initial combination therapy is cost effective, also when the uncertainty in the model inputs were considered in probabilistic sensitivity analysis. That strategy yields more QALYs but also saves cost in absolute terms. The difference in cost between the two strategies is almost €4000 over 5 years. The difference in accrued QALYs is smaller, 0.0325. Uncertainty around estimated costs and effects was small. The ICER is negative because initial combination therapy is a dominant strategy. These results were shown to be robust in the PSA. Figure 3 shows the ICER scatterplot for the probabilistic sensitivity results. This ICER plane shows the incremental cost-effectiveness of initial combination therapy strategy as compared to step-up therapy. It shows that a large proportion of trials is in the southeast quadrant, with positive incremental utility, and negative incremental costs.

**Table 2:** Probabilistic Sensitivity Analysis results. QALY: Quality-Adjusted Life Years, ICER: Incremental Cost-Effectiveness Ratio

	Step-up	Initial Combination
Mean costs (€)	€20,163.81	€16,267.15
2.5–97.5% interval	16,588.46–23,780.33	11,534.72–21,366.87
Mean utility (QALY)	3.515	3.548
2.5–97.5% interval	2.467–4.598	2.44–4.71
ICER	–	−119,897 (Dominating)

## Discussion

Treating to the target of remission of recent onset RA with combination therapy versus step-up therapy with disease modifying anti rheumatic drugs is more effective (in terms of EQ-5D utility) and less costly. According to the results in the current study, early-onset RA patients being treated with initial combination therapy accrue higher utility and lower costs over five years as compared to patients being treated with step-up therapy. A negative ICER is ambiguous, as it can indicate that the intervention is more costly while less effective (meaning it is dominated) or that it is less costly and more effective, hence, a dominant strategy. The negative ICER in this study shows that the second strategy, initial combination therapy, is both less costly and more effective, thus making it an absolute preferable option and dominant strategy over step-up therapy. The difference in accrued QALYs between the two modelled strategies is relatively small at 0.0325 over 5 years. This could be due to the fact that the strategies are both treating to the target of remission, thus have the same therapeutic goals.

Overall, there are more patients in remission in initial combination therapy strategy than in the step-up therapy strategy at each time point. This reduces health costs, increases utility, and medication can be tapered lowering medication costs. From a health-economic perspective, physicians should thus prefer initial combination over step-up therapy to treat to the target of remission strategy.

In this present study data obtained from two cohort studies in patients with early RA to project 5-year outcomes of two different treat-to-target strategies were used. Clinical outcomes obtained in these cohorts were previously compared [29], which showed more remissions at 6 months with initial combination therapy than step-up therapy. The results of this current modelling study suggest that this trend continues with higher remission percentages across all time points. The present study is among the first modelling studies to specifically evaluate outcomes of early RA patients who are enrolled in a tight-control strategy immediately upon diagnosis. In previous modelling studies in RA, it is usually assumed that the status of patients progressively deteriorates, which is typically modelled as decreasing Health Assessment Questionnaire Disability index (HAQ-DI)-scores over time. However, a pattern of deteriorating status is not consistent with studies describing 5–10 year outcomes of early RA cohort studies in which a tight control protocol was used, and was therefore not considered appropriate for the current study. In reports describing long term outcomes of such cohorts, HAQ-DI scores typically follow the same pattern as the DAS28 scores, with initial high disability followed by a prolonged (5–10 years) period of stable low disability according to HAQ-DI [30–36].

Schipper et al. first adapted the Welsing et al. Markov model [15] that was also used in his study. In the paper by Schipper et al. [14], the model reaches an equilibrium at 2.5 years, with no further transitions occurring after that time point. This presumably happened because the majority of the patients were absorbed in the ‘sustained remission’ state. In the present study, the Schipper et al.’s version of the model was adapted in several ways to better reflect the T2T based treatment strategies, as well as the disease course of RA, that is characterized by recurrent flares. Simulated patients were now able to reach sustained remission and have their medication tapered, but in case of a flare they were also able to return to their last effective medication. Remission was no longer an absorbing state and individual simulated patients’ disease course fluctuates over time, as in real-life. In line with studies on stopping and tapering of TNFi’s, in this model, biological-free remission has become an option for RA patients. Despite these adjustments, Schipper et al. have found comparable results over the 5-year modelling period. Accrued QALY’s over 5 years are slightly lower, which could be explained by the different discount rate for utilities that the authors used. Schoels et al., [3] who performed a literature review of economic aspects of treatment sequences in RA have focussed on step-up therapy, similar to the one employed in this study, as the most cost-effective option versus employing TNF-i’s in an earlier stage of the disease process. This paper confirms this notion and extends it to longer-term cost-effectiveness.

A major strength of this study is the use of real world daily clinical practice data from recent onset RA patients. The usage of daily clinical practice data assures that patients were not selected and the study group fully represents all types of RA patients and suggests that the results readily translate to clinical practice settings in which early RA patients will be treated to target, upon diagnosis. Moreover, projected disease activity outcomes from the modelled cohorts were shown to closely approximate real world outcomes of patients seen in practice, as displayed in Fig. 2. A limitation of this study is that due to a lack of data on productivity loss and employment for all patients, there was no possibility to collect out-of-pocket expenses or use the friction cost method, which should be considered when comparing our results to results obtained in other cost-effectiveness studies in this patient population.

This study analysed data from the Dutch societal perspective. Generalizing the results to other European countries should be handled carefully as medication list prices can vary across European countries, even in spite of the external reference pricing system that many EU members apply [37]. Additionally, the EQ-5D tariff, which is used to calculate the utility score from the EQ-5D questionnaire, varies per country. However, EULAR recommendations for T2T management apply to all European countries. Additionally, clinical features, like the DAS28 patterns, are not likely to vary per country. All in all, the model could be adapted to give an accurate representation of a different (European) country by adjusting the medication prices and the EQ-5D tariff.

## Conclusion

In summary, the results of this study suggest that treating recently diagnosed RA-patients to the target of remission according to this strategy of initial combination therapy not only results in more patients in beneficial states of disease activity (remission or low disease activity) compared with step-up therapy, but also at lower costs.

## Additional file

Additional file 1: Supplemental Material - Baseline characteristics. Baseline Characteristics of DREAM Remission Induction Cohorts I and II. Description of data: Baseline Characteristics table of the two cohorts (RIC I and RIC II) from which data was used in this study. It includes sex, age, mean DAS28, tender joint count, swollen joint count, median erythrocyte sedimentation rate, median c-reactive protein, median health assessment questionnaire disability index, short-form 36 health survey, mean physical component summary, mean mental component summary, percentage rheumatoid factor positive, percentage anti-cyclic citrullinated peptide positive, mean body mass index. (DOCX 21 kb)

## Abbreviations

ACR: American College of Rheumatology
anti-TNF: Anti-tumour necrosis factor drugs
bDMARD: Biological disease modifying anti-rheumatic drugs
csDMARD: Conventional synthetic disease modifying anti-rheumatic drugs
DAS28: Disease activity score in 28 joints using erythrocyte sedimentation rate
DMARDs: Disease modifying anti-rheumatic drugs
DREAM: Dutch RhEumatoid Arthritis Monitoring registry
EQ-5D: EuroQol five dimensional questionnaire
EULAR: European League Against Rheumatism
HAQ-DI: Health Assessment Questionnaire Disability index
HCQ: Healthcare Consumption Questionnaire
ICER: Incremental cost-effectiveness ratio
LDA: Low disease activity
MTX: Methotrexate
PSA: Probabilistic sensitivity analysis
QALY: Quality adjusted life year
RA: Rheumatoid arthritis
RIC I: Remission Induction Cohort I
RIC II: Remission Induction Cohort II
RIC: Remission Induction Cohort
T2T: Treat-to-Target
WTP: Willingness-to-Pay threshold
