# Supplementary material for: Cost-effectiveness of different treat-to-target strategies in rheumatoid arthritis: results from the DREAM registry
Source: BMC Rheumatol. 2019 Apr 29;3:16. doi: 10.1186/s41927-019-0064-9 (PMC6487515; doi:10.1186/s41927-019-0064-9)
Supplement: Supplementary file 1 — Supplemental Material - Baseline characteristics. Baseline Characteristics of DREAM Remission Induction Cohorts I and II. Description of data: Baseline Characteristics table of the two cohorts (RIC I and RIC II) from which data was used in this study. It includes sex, age, mean DAS28, tender joint count, swollen joint count, median erythrocyte sedimentation rate, median c-reactive protein, median health assessment questionnaire disability index, short-form 36 health survey, mean physical component summary, mean mental component summary, percentage rheumatoid factor positive, percentage anti-cyclic citrullinated peptide positive, mean body mass index. (DOCX 21 kb) [file 41927_2019_64_MOESM1_ESM.docx]

|  | **RIC I (N = 509)** | **RIC II (N= 381)** |
| --- | --- | --- |
| **Female, sex n (%)** | 321 (63.1%) | 227 (59.6%) |
| **Age, mean ± SD years** | 58.5 ± 14.5 | 59.8 ± 13.7 |
| **DAS28, mean ± SD** | 4.3^k^ ± 1.5 | 4.1^c^ ± 1.6 |
| **Number of TJC, median (IQR)** | 3.0 ( 1.0 – 7.0) | 2.0 (0.0 – 6.0) |
| **Number of SJC, median (IQR)** | 5.0 (2.0 – 9.0) | 3.0 (1.0 – 8.0) |
| **ESR (mm/h), median (IQR)** | 22.0^i^ (11.0 – 38.0) | 20.0 ^a^ (9.0 – 36.0) |
| **CRP (mm/h), median (IQR)** | 10.0^j^ (5.0 – 20.0) | 7.0^b^ (3.0 - 18.0) |
| **Anti-CCP positive, n(%)** | 253^l^ (58.2%) | 133^d^ (58.3%) |
| **RF positive, n (%)** | 355^m^ (71.9%) | 145^e^ (59.2%) |
| **SF36-PCS, mean ± SD** | 36.8^n^ ± 8.7 | 38.6^f^ ± 9.6 |
| **SF36-MCS, mean ± SD** | 48.1^o^ ± 11.7 | 46.5^f^ ± 12.2 |
| **HAQ-SDI, median (IQR)** | 1.3^p^ ( 0.6 – 1.9) | 0.8^g^ (0.3 – 1.4) |
| **BMI kg/m^2^, mean ± SD** | 26.6^q^ ± 4.9 | 26.2^h^ ± 4.5 |

^a^ = 377; ^b^ = 362; ^c^ = 367; ^d^ = 228; ^e^ = 245; ^f^ = 249; ^g^ = 253; ^h^ = 124; ^i^ = 500; ^j^ = 486; ^k^ = 500; ^l^ = 435; ^m^ = 494; ^n^ = 401; ^o^ = 401; ^p^ = 478; ^q^ =383

*DAS28 = Disease Activity Score in 28 joints; ESR = erythrocyte sedimentation rate; CRP = C-reactive protein; TJC = tender joint count; SJC = swollen joint count; HAQ-SDI = Health Assessment Questionnaire disability index (standard scoring); SF-36 = Short-Form 36 health survey (version 2); PCS = physical component summary; MCS = mental component summary; RF = rheumatoid factor; Anti-CCP = anti-cyclic citrullinated peptide; BMI = body mass index; RIC = Remission Induction Cohort*
